# Possibility of selection against mtDNA mutations in tumors

**DOI:** 10.1186/1476-4598-4-36

**Published:** 2005-09-13

**Authors:** M Khaidakov, RJ Shmookler Reis

**Affiliations:** 1 Department of Geriatrics, University of Arkansas for Medical Sciences, John McClellan Veterans Medical Center, 4300 West 7^th ^Street, Little Rock, AR 72205, USA

**Keywords:** tumorigenesis, mitochondrial DNA, mutations

## Abstract

Several studies of tumors have revealed substantial numbers of clonally expanded somatic mutations in mitochondrial DNA (mtDNA), not observed in adjacent intact tissues. These findings were interpreted as indicating the involvement of mtDNA mutations in tumorigenesis. Such comparisons, however, ignore an important confounding factor: the monoclonal origin of tumors as opposed to the highly polyclonal nature of normal tissues. Analysis of recently published data on the incidence of somatic mutations in nontumor monoclonal cells suggests that, contrary to the prevailing view, the process of tumorigenesis may be accompanied by active selection against detrimental mtDNA mutations.

Accumulation in mutations in mtDNA, leading to an impairment of mitochondrial function, has been implicated in the etiology of aging [1,2] and of several degenerative pathologies including Parkinson's [3], Alzheimer's diseases [4], and diabetes [5]. Similarly, mtDNA mutation is thought to be involved in tumorigenesis based on the presence of novel hetero- and homoplasmies in a number of neoplasms [6]. In the case of degenerative diseases, intracellular clonal expansion of detrimental mtDNA mutations could play a causative role, resulting in cell loss and/or significant functional impairment of affected cells. This logic is not likely to apply to tumorigenesis, and it is not entirely clear at what stage of tumorigenesis a deficiency in aerobic metabolism would confer a selective advantage.

Several different scenarios, linking mtDNA mutations to tumorigenesis, can be envisioned. Cells with an elevated mtDNA mutational load may be more susceptible to carcinogenesis due to increased production of ROS by a dysfunctional electron transport chain, which may in turn promote mutagenesis of nuclear genes. On the other hand, cells with relatively intact oxidative phosphorylation would have enhanced prospects for survival. It is also possible that carcinogenesis is influenced to a larger extent by accumulation of mtDNA mutations in organs distal to the tissue of origin, by creating a systemically permissive environment for development of tumors.

In a number of studies on mtDNA mutations derived from diverse tumors [7-20], the percentage of samples with clonally expanded mtDNA mutations ranged from 27% to almost 80%, averaging 54 ± 5% (mean ± SE; see Suppl. Table 1). These findings led investigators to the conclusion that mtDNA mutations are either more common in carcinogenesis, or in some way predispose to it [8,21]. It is not possible, however, to infer the functional importance of mitochondrial DNA mutations to tumorigenesis from these comparisons of tumors to adjacent normal tissue. Such studies are confounded by the well-established monoclonal origin of tumors in contrast to the highly polyclonal derivation of normal tissues. Therefore, while the occurrence of hetero- or homoplasmic mutations to mitochondrial DNA is easily detectable in tumor cells, low-level heteroplasmy remains undetected by prevailing methods in essentially all normal tissues. In addition, tumor cells are generally more mitotically active than normal diploid cells [22] and, as clonal expansion of mutations is thought to result from the random distribution of mutations over multiple replicative cycles [23], tumors could display higher rates of subclonal development and detection of new heteroplasmies, for this reason alone. Consequently, the validity of direct comparisons between tumor and adjacent normal tissues is questionable, when such studies fail to address the frequency of mitochondrial DNA mutations in individual normal cells or clones of normal cells that have undergone a comparable expansion.

This type of analysis has been recently performed in both dividing and non-dividing normal cells [24-27]. In a study on individual buccal cells and myocytes, direct sequencing of the control region (a non-coding 1121 bp fragment of mtDNA containing several replicational and transcriptional control elements) revealed clonally expanded mutations in 36% of both cell types from older individuals, whereas cells from younger subjects were free from homoplasmies [24]. In another study from the same group, homoplasmy in the D-loop was detected in 20% (5/24) of buccal cells [25], thus demonstrating that clonal expansion is not a tumor-specific phenomenon. Using available data on tumor dynamics the authors estimated the occurrence of clonal expansions in the entire mtDNA of tumors at 58%, and noted the similarity of this frequency to reported empirical values [8,21].

Our analysis of data from available literature (Fig. 1, Additional file 1, Table 1) supports the above argument [23], that normal cells and tumors have very similar frequencies of clonally expanded mutations in the D-loop (24 ± 5 *vs*. 30 ± 5%) and in the entire mitochondrial genome (48 ± 12% *vs*. 54 ± 5%). However, analysis of the mutation distribution within mtDNA in tumors and normal cells reveals a more complex pattern, in which the similar totals hide very disparate components. Nearly half of the mutations found in tumors (47 ± 7%) are located in the control region (Fig. 2, Additional file 1, Table 2). The remaining mutations comprise 13% silent substitutions in protein-coding sequence, 21% missense/nonsense changes in the same regions, and 19% changes in sequences templating mitochondrial RNAs. The only fully comparable normal-tissue dataset available is derived from the analysis of complete mtDNA sequences in 60 colonic crypts from five healthy individuals [26]. Colonic crypts are thought to be of essentially clonal derivation [28], and thus provide an appropriate control for cancer cells. Compared to cancer cells, the incidence of D-loop mutations in mtDNA of colonic crypts was more than 3-fold lower (*P *< 0.0002), whereas the percentage of both mutations in RNA sequences and missense/nonsense mutations in protein coding sequences was about 75% higher.

**Figure 1:**
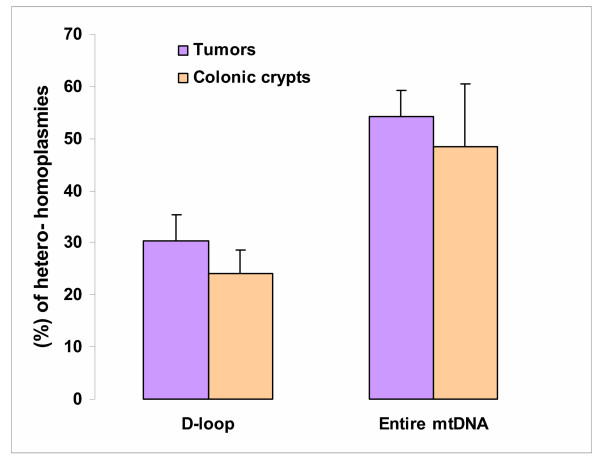
Incidence of somatic mutations in tumors and normal cells (The datasets from colonic crypts [26] have been modified in order to eliminate bias introduced by preferential analysis of COX negative crypts; for this purpose, all functionally detrimental mutations involving COX subunits in COX-negative crypts were removed from consideration).

**Figure 2:**
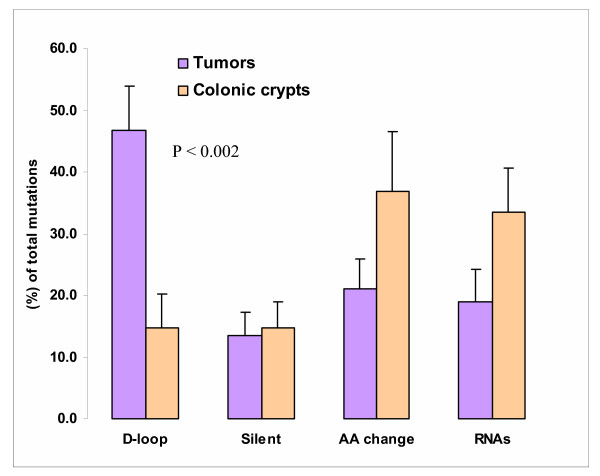
Distribution of mtDNA mutations in tumors and normal cells. (*P *values refer to *t*-test comparisons between tumor and nontumor cells; detailed information is provided in Additional file 1, Table 2).

The incidence of somatic mutations in the control region of mtDNA is similar for tumors and crypt cells, consistent with these cells having comparable mitotic activity. On the other hand, the ratio of mutation frequencies (per 1 kb) in the D-loop, versus the rest of mtDNA, is much higher in tumors (13.5) than in normal epithelial cells (2.4), suggesting a rather different distribution of mutations or (more likely) very different selective pressures acting on these cell types with respect to mitochondrially encoded subunits. These data support strong selection against detrimental mtDNA mutations in tumor cells, implying that successful tumorigenesis requires intact mitochondria.

## Supplementary Material

Additional file 1 Numerical data on incidence and distribution of mtDNA mutations in tumors and normal cells. Click here for file
