# Supplementary material for: Possibility of selection against mtDNA mutations in tumors
Source: Mol Cancer. 2005 Sep 13;4:36. doi: 10.1186/1476-4598-4-36 (PMC1232865; doi:10.1186/1476-4598-4-36)
Supplement: Additional file 1 — Numerical data on incidence and distribution of mtDNA mutations in tumors and normal cells. [file 1476-4598-4-36-S1.doc]

**Additional file 1.** Numerical data on Incidence and distribution of mtDNA mutations in tumors and normal cells.

Table 1. mtDNA somatic mutations found in tumors and normal cells

| Tissue/Organ | **# Total Samples** | **# with SM** | **% with**  **SM** | **# Only**  **In D-loop** | **% in**  **D-loop** | **Reference** |
| --- | --- | --- | --- | --- | --- | --- |
| Tumors | | | | | | |
| Leukaemia | 24 | 9 | 37.5 | 2 | 8.3 | He *et al.*, 2003 |
| Breast cancer | 19 | 14 | 73.7 | 12 | 63.2 | Tan *et al.*, 2002 |
| Oral carcinomas | 18 | 14 | 77.8 | 12 | 66.7 | Tan *et al.*, 2004 |
| Ovarian cancer | 10 | 6 | 60.0 | 2 | 20.0 | Liu *et al.*, 2001 |
| Renal cell carcinoma | 8 | 5 | 62.5 | 1 | 12.5 | Nagy *et al.*, 2002 |
| Bladder cancer | 14 | 9 | 64.3 | 4 | 28.6 | Fliss *et al.*, 2000 |
| Head and neck ca. | 13 | 6 | 46.2 | 3 | 23.1 | Fliss *et al.*, 2000 |
| Lung cancers | 11 | 3 | 27.3 | 2 | 18.2 | Fliss *et al.*, 2000 |
| Medulloblastoma | 15 | 6 | 40.0 | 6 | 40.0 | Wong *et al.*, 2003 |
| Breast cancer | 18 | 11 | 61.1 | 7 | 38.9 | Parrella *et al.*, 2001 |
| Lung cancers | 58 | 36 | 62.1 | n/a | n/a | Jin *et al.*, 2002 |
| Head and neck ca. | 11 | 4 | 36.4 | 1 | 9.1 | Chun-Yang *et al*., 2004 |
| Colorectal cancers | 77 |  |  | 7 | 9.1 | Hibi *et al.*, 2001a |
| Esophageal cancer | 37 |  |  | 2 | 5.4 | Hibi *et al.*, 2001b |
| Esophageal cancer | 20 |  |  | 8 | 40.0 | Miyazono *et al.*, 2002 |
| Stomach cancer | 45 |  |  | 2 | 4.4 | Tamura *et al.*, 1999 |
| Liver carcinoma | 50 |  |  | 17 | 34.0 | Okochi *et al.*, 2002 |
| MeanSE | |  | **54.1** |  | **30.3** |  |
|  |  |  | **4.6** |  | **5.2** |  |
| **Normal cells** | | | | | | |
| Colonic crypt cells | 12 | 9 | 66.7 | 1.0 | 8.3 | Taylor *et al.*, 2003 |
|  | 12 | 8 | 58.3 | 1.0 | 8.3 |  |
|  | 12 | 7 | 58.3 | 3.0 | 25.0 |  |
|  | 12 | 7 | 58.3 | 1.0 | 8.3 |  |
|  | 12 | 0 | 0.00 | 0.0 | 0.0 |  |
| Buccal epithelium | 12 |  |  | 5.0 | 41.7 | Nekhaeva *et al.*, 2002 |
|  | 12 |  |  | 4.0 | 33.3 |  |
|  | 12 |  |  | 6.0 | 50.0 |  |
| Myocytes | 12 |  |  | 2.0 | 16.7 |  |
|  | 12 |  |  | 4.0 | 33.3 |  |
|  | 12 |  |  | 9.0 | 75.0 |  |
| Buccal epithelium | 24 |  |  | 5.0 | 20.8 | Coller *et al.*, 2001 |
| MeanSE | |  | **48.3** |  | **24.0** |  |
|  |  |  | **12.2** |  | **5.0** |  |

“SM” – total samples with somatic mutations; “in D loop” – samples with somatic mutations observed in the D-loop region; n/a – no data available;

**Table 2. Distribution of somatic mutations in tumors and individual normal cells.**

| **DL** | **Silent** | **AA change** | **rRNAs** | | **Total** | **silent** | **AA change** | **rRNAs** | **D-loop** | **(%) silent*** | **Reference** |
| --- | --- | --- | --- | --- | --- | --- | --- | --- | --- | --- | --- |
| **Location** | | | | | | **(%) of total mutations** | | | |  |  |
| Tumors | | | | | | | | | | |  |
| 2 | 2 | 4 | 1 | | 9 | 22.2 | 44.4 | 11.1 | 22.2 | 33.3 | He *et al.*, 2003 |
| 22 | 3 | 1 | 1 | | 27 | 11.1 | 3.7 | 3.7 | 81.5 | 75.0 | Tan *et al.*, 2002 |
| 20 | 2 | 4 | 0 | | 26 | 7.7 | 15.4 | 0.0 | 76.9 | 33.3 | Tan *et al.*, 2004 |
| 2 | 0 | 1 | 3 | | 6 | 0.0 | 16.7 | 50.0 | 33.3 | 0.0 | Liu *et al.*, 2001 |
| 1 | 1 | 2 | 2 | | 6 | 16.7 | 33.3 | 33.3 | 16.7 | 33.3 | Nagy *et al.*, 2002 |
| 5 | 9 | 2 | 3 | | 19 | 47.4 | 10.5 | 15.8 | 26.3 | 81.8 | Fliss *et al.*, 2000 |
| 5 | 0 | 2 | 1 | | 8 | 0.0 | 25.0 | 12.5 | 62.5 | 0.0 | Fliss *et al.*, 2000 |
| 3 | 0 | 0 | 3 | | 6 | 0.0 | 0.0 | 50.0 | 50.0 | 0.0 | Fliss *et al.*, 2000 |
| 11 | 2 | 2 | 3 | | 18 | 11.1 | 11.1 | 16.7 | 61.1 | 50.0 | Wong *et al.*, 2003 |
| 7 | 2 | 3 | 0 | | 12 | 16.7 | 25.0 | 0.0 | 58.3 | 40.0 | Parrella *et al.*, 2001 |
| 1 | 1 | 3 | 1 | | 6 | 16.7 | 50.0 | 16.7 | 16.7 | 25.0 | Present study |
| MEANSE | | | | | | **13.6** | **21.4** | **19.1** | **46.0** | **37.2** |  |
|  |  |  |  |  |  | **4.1** | **4.8** | **5.4** | **7.2** | **8.5** |  |
| **Normal cells** | | | | | | | | | | |  |
| 1 | 1 | 6 | | 4 | 12 | 8.3 | 50.0 | 33.3 | 8.3 | 14.3 | Taylor *et al.*, 2003 |
| 1 | 4 | 3 | | 7 | 15 | 26.7 | 20.0 | 46.7 | 6.7 | 57.1 |  |
| 3 | 1 | 2 | | 4 | 10 | 10.0 | 20.0 | 40.0 | 30.0 | 33.3 |  |
| 1 | 1 | 4 | | 1 | 7 | 14.3 | 57.1 | 14.3 | 14.3 | 20.0 |  |
| MEANSE | | | | | | **14.8** | **36.8** | **33.6** | **14.8** | **31.2** |  |
|  |  |  |  |  |  | **4.1** | **9.8** | **7.0** | **5.3** | **9.5** |  |

(*) – percent of silent mutations in the protein coding sequence: DL – D-loop mutations; AA change – missense

mutations altering the encoded amino acid.
